# Supplementary material for: CEACAM1 as a mediator of B-cell receptor signaling in mantle cell lymphoma
Source: Nat Commun. 2025 May 29;16:4967. doi: 10.1038/s41467-025-60208-3 (PMC12120064; doi:10.1038/s41467-025-60208-3)
Supplement: Supplementary file 1 — Supplementary Information [file 41467_2025_60208_MOESM1_ESM.docx]

**Supplementary Information**

**CEACAM1 as a Mediator of B-cell Receptor Signaling in Mantle Cell Lymphoma**

Serene Xavier *et al.*

Corresponding author: Vu N. Ngo, vngo@coh.org

This document contains:

Supplementary Fig. 1 to 13

Other Supplementary Information in this study include:

Supplementary Data 1 to 9

Supplementary Software: Quantitative image analysis scripts

Source Data Fig. 1 to 7

Source Data Supplementary Fig. 1 to 12

**Supplementary Figures**

**
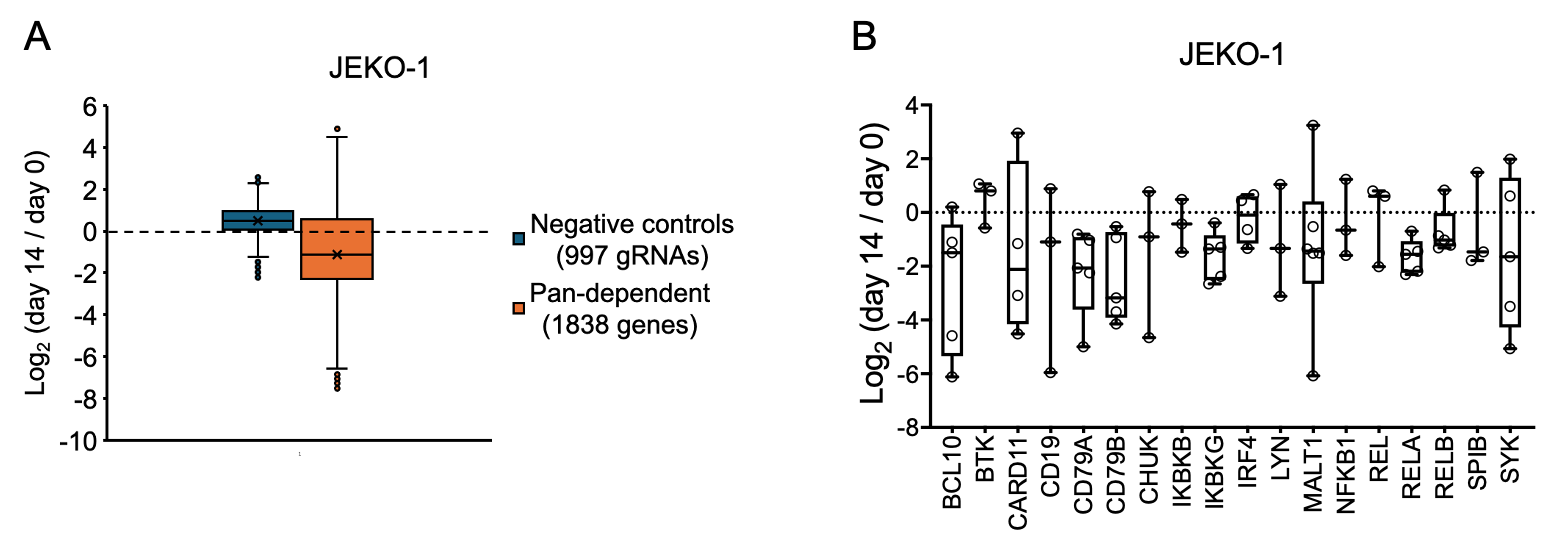
**

**Supplementary Fig. 1: Results of controls in JEKO-1 CRISPR library screen.** Box plots show Log2 fold-change (LFC) of negative control gRNAs and pan-dependent genes (A) and genes in the BCR and NFκB signaling pathways (B).

**
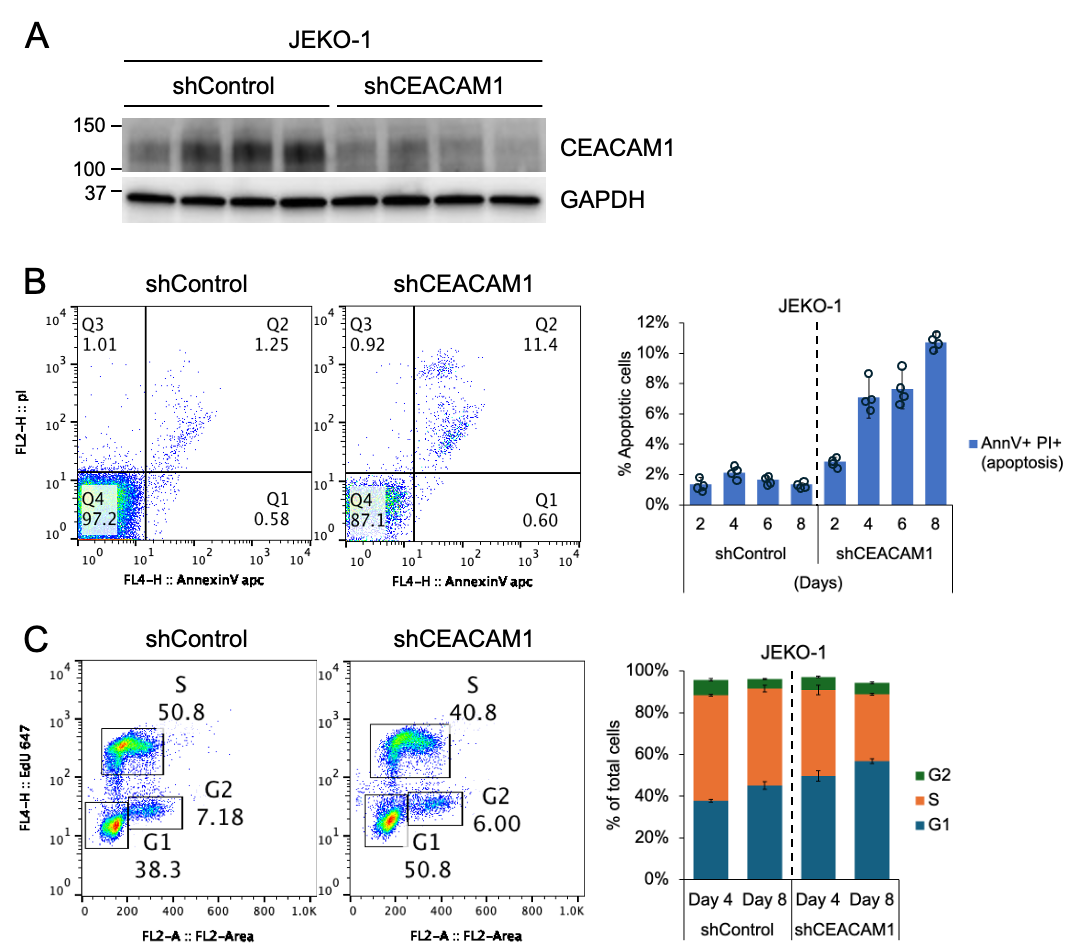
**

**Supplementary Fig. 2: Effects of CEACAM1 knockdown on apoptosis and cell cycle. A**, Immunoblots show CEACAM1 depletion by shRNA transduction in JEKO-1 cells compared to control in four independent experiments. **B**, Representative flow cytometry plots of JEKO-1 cells transduced with control or CEACAM1 shRNA and stained for Annexin V and propidium iodide (PI). *Right panel*, quantification of % apoptotic cells (Annexin V+PI+) from four independent experiments. **C**, Representative cell cycle analysis of JEKO-1 cells transduced with control or CEACAM1 shRNA. *Right panel*, quantification of each cell cycle phase for the indicated times post-transduction from four independent experiments.


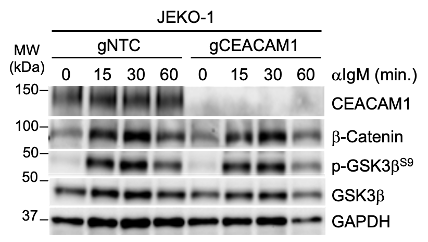

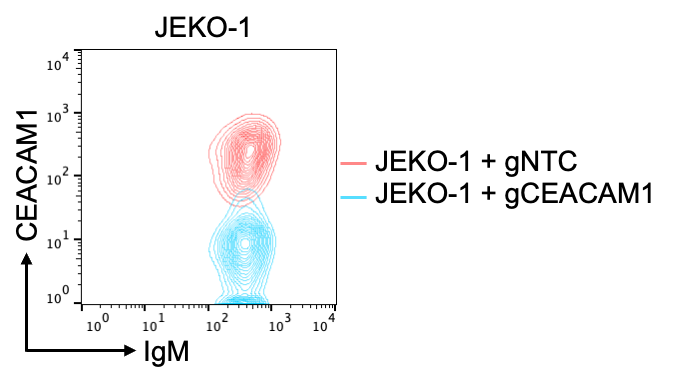

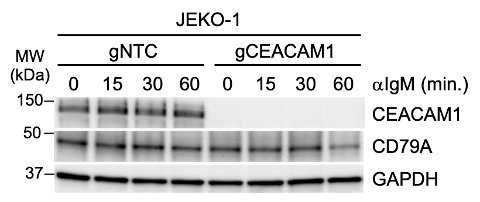

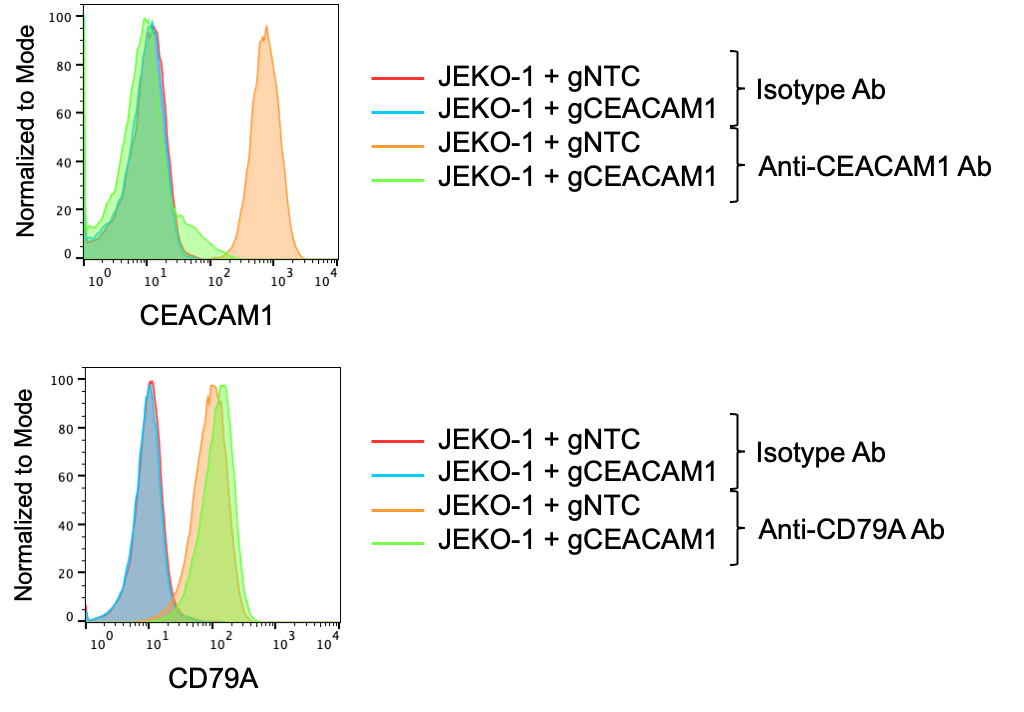


D

C

B

A

**Supplementary Fig. 3: Effects of CEACAM1 knockout on CD79A and IgM expression and beta-catenin signaling. A,** JEKO-1 cells were transduced with control (gNTC) or CEACAM1 gRNA (gCEACAM1) and analyzed for CEACAM1 and CD79A expression by flow cytometry. **B,** JEKO-1 cells were transduced as described in (A) followed by stimulation with 2 ug/ml of anti-IgM for the indicated time and immunoblotting with indicated antibodies. **C,** Contour flow cytometry plot comparing IgM expression between gNTC- or gCEACAM1-transduced JEKO-1 cells. **D,** JEKO-1 cells were transduced as described in (A) followed by stimulation with 2 ug/ml of anti-IgM for the indicated time and immunoblotting with indicated antibodies. Representative FACS plots in (A) and (C) and immunoblots in (B) and (D) are from two independent experiments.

**
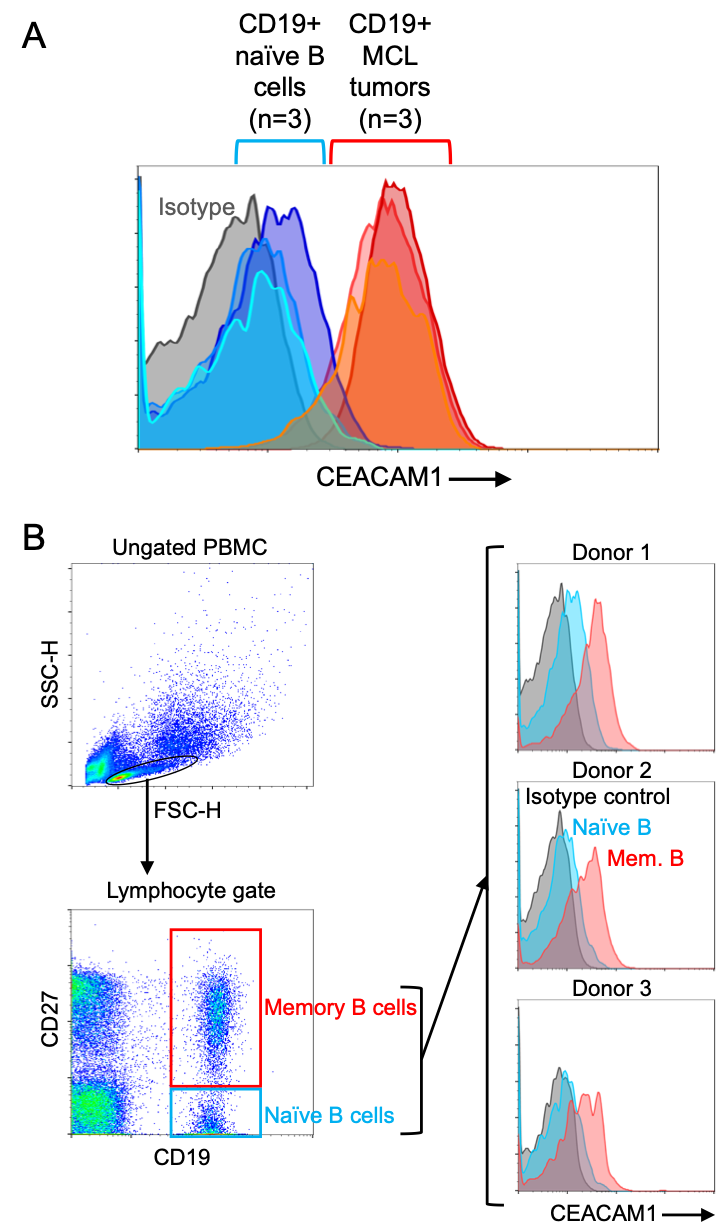
**

**Supplementary Fig. 4: CEACAM1 expression in MCL and normal B cells**. **A,** Representative FACS plots show CEACAM1 expression on CD19^+^ naïve B cells from three healthy donors were compared with three CD19^+^ MCL samples by flow cytometry. Isotype, negative control using isotype antibody on naïve B cells. **B,** Gating scheme for flow cytometric analysis of CEACAM1 expression on naïve and memory B cells from peripheral blood mononuclear cells from three healthy donors as shown in (**A**).

**
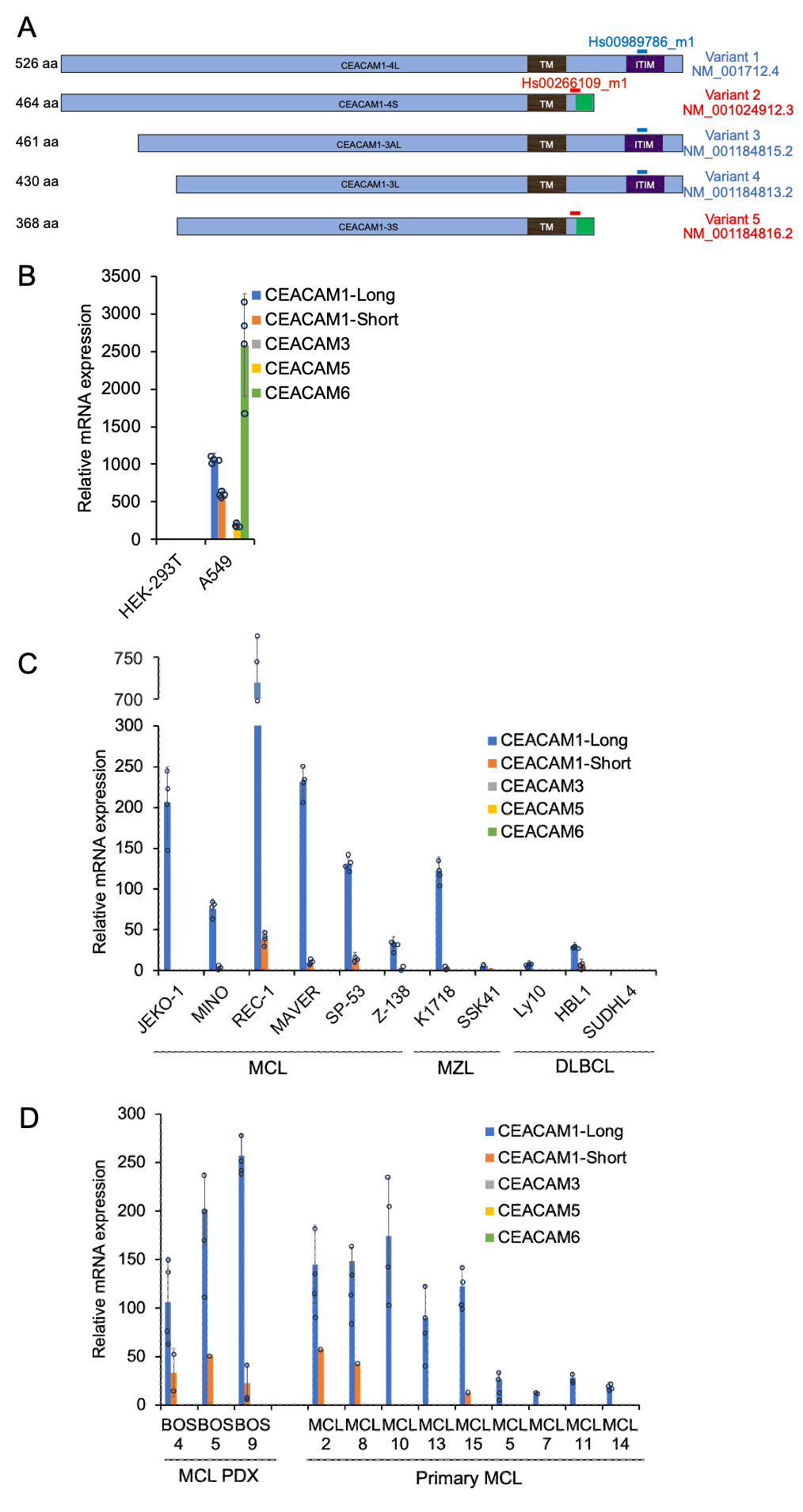
**

**Supplementary Fig. 5: CEACAM1 expression in lymphoma cells by qPCR analysis**. **A,** Diagrams of CEACAM1 coding variants and corresponding NCBI gene accession numbers show varying amino acid (aa) lengths and locations of Taqman probes. TM, transmembrane. ITIM, immunoreceptor tyrosine based inhibitory motif. The green area indicates the distinct amino acid sequence resulted from alternative splicing in the cytoplasmic tail of the short isoforms (variants 2 and 5). **B,** Validation of Taqman probes*.* Expression analysis by qPCR using Taqman probes specific for each CEACAM molecules was performed on mRNA harvested from the human lung cancer cell line A549 and HEK-293T cells as positive and negative control, respectively. Shown are the means of mRNA expression levels after normalization to GAPDH signals from 4 independent amplification experiments. Error bars, SD. **C,D,** qPCR analysis of mRNA expression levels for indicated CEACAM molecules in indicated cell lines, MCL PDX or primary MCL samples using specific Taqman probes validated in (**B**). Shown are the means of mRNA expression levels after normalization to GAPDH signals from 4 independent amplification experiments. Error bars, SD.


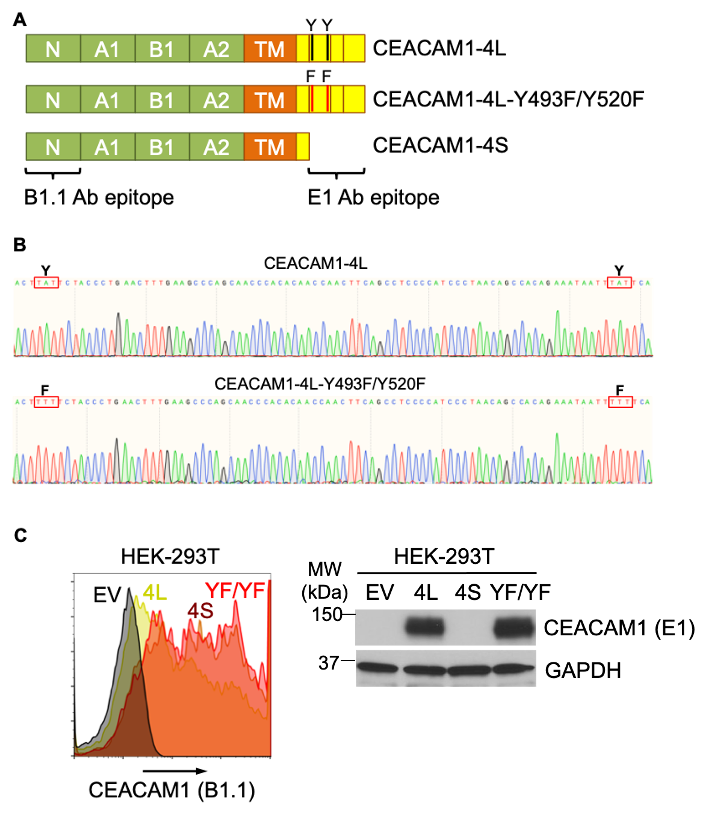


**Supplementary Fig. 6: Validation of CEACAM1 mutant constructs. A,** Diagram of wild type CEACAM1 (CC1-4L), mutant ITIM (CC1-4L-Y493F/Y520F) and deleted cytoplasmic tail (CC1-4S). Epitopes that are recognized by specific CEACAM1 antibody clones (B1.1 or E1) are indicated. **B,** Chromatograms show mutations at the ITIM tyrosines of the CC1-4L-Y493F/Y520F construct. **C,** *Left panel.* Representative FACS plots show CEACAM1 surface expression in HEK-293T cells using the CEACAM1 antibody clone B1.1 from two independent experiments. *Right panel.* Representative immunoblots show CEACAM1 expression in HEK-293T cells using the CEACAM1 antibody clone E1 from two independent experiments. EV, empty vector; 4L, CEACAM1-4L; 4S, CEACAM1-4S; YF/YF, CEACAM1-4L-Y493F/Y520F.


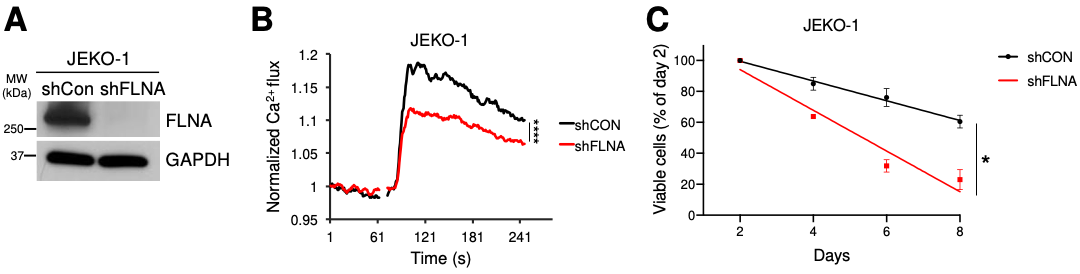


**Supplementary Fig. 7: FLNA is required for BCR signaling and survival in MCL**. **A,** JEKO-1 cells transduced with control or FLNA shRNA and analyzed by immunoblotting with indicated antibodies. **B,** Ca^2+^ flux signals in indicated control or FLNA-knockdown JEKO-1 cells following anti-IgM stimulation (1μg/mL). **** P<0.0001 by two-way ANOVA. **C,** Effects of FLNA knockdown on cell survival. JEKO-1 cells were transduced with control or FLNA shRNA co-expressing GFP. Viable, GFP+ cells were monitored over time by flow cytometry. Shown are the means of GFP+ fractions compared to day-2 samples from at least two independent experiments. Error bars, SD. * p<0.05 by two-way ANOVA.

**Supplementary Fig. 8: CEACAM1 is required for optimal assembly of lipid rafts.** Control (gNTC) or CEACAM1 knockout (gCEACAM1) JEKO-1 cells were stimulated with 2 μg/ml anti-IgM antibody for 2 min. Shown are representative confocal immunofluorescence images (acquired with a 20x/0.8NA objective) of control and IgM-stimulated cells co-stained with anti-FLNA (green) and anti-LYN (red) antibodies followed by nuclear staining with DAPI (blue) from three independent experiments. Scale bar, 10 μm.

**
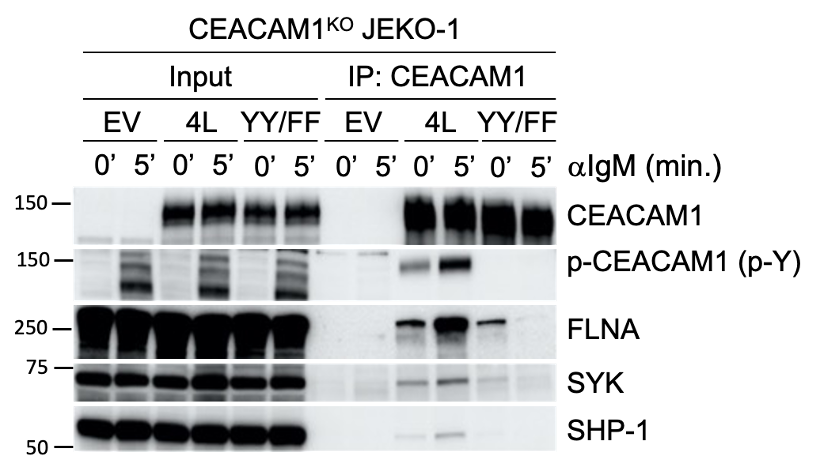
**

**Supplementary Fig. 9: CEACAM1 interactions with signaling components require the intact ITIM tyrosine residues**. CEACAM1-knockout (KO) JEKO-1 cells were transduced with either empty vector (EV), WT (4L), or CEACAM1-4L-Y493F/Y520F (YY/FF) constructs, stimulated with 2 μg/ml anti-IgM antibody for 5 min, followed by CEACAM1 immunoprecipitation and immunoblotting with indicated antibodies. One percent of total lysates was used as input control. p-Y, anti-phosphotyrosine antibody clone 4G10. Shown are representative immunoblots from two independent experiments.

**
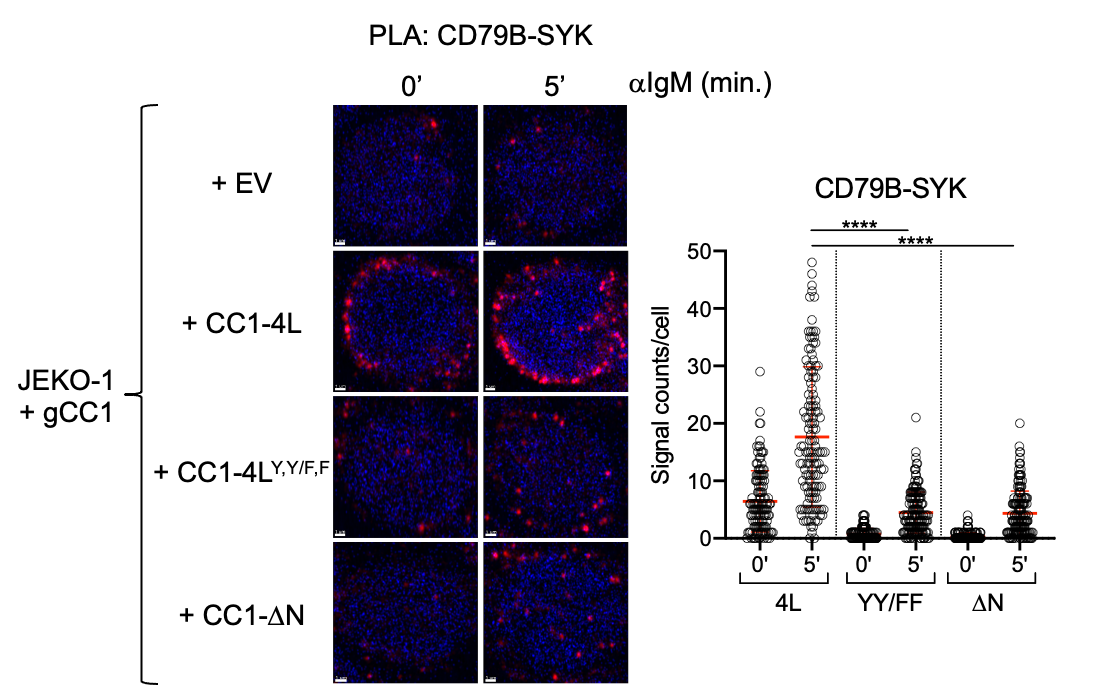
**

**Supplementary Fig. 10: Impact of CEACAM1’s structural features on the interaction between CD79B and SYK**. CEACAM1-knockout JEKO-1 cells (+ gCC1) were transduced with either empty vector (EV), WT (CC1-4L), CEACAM1-4L-Y493F/Y520F (CC1-4L^Y,Y/F,F^), or N-domain truncated (CC1-ΔN) CEACAM1 constructs, stimulated with 2 μg/ml anti-IgM for 5 min, followed by proximity ligation assays (visualized as red dots) between CD79B and SYK (*left panel*). *Right panel*, Representative quantitation of PLA signals shown in *left* for 100-200 cells on average from two independent experiments using QuPath software. Error bars indicate means with S.D. **** P<0.0001 by 2-sided Mann-Whitney U test.

**
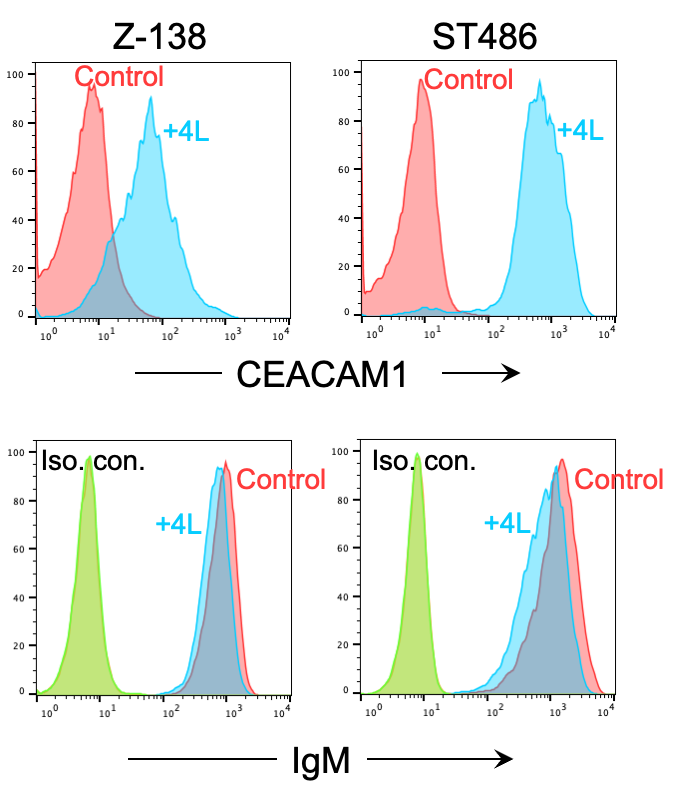
**

**Supplementary Fig. 11: Effects of CEACAM1 expression on IgM surface levels.** Z-138 and ST486 cells were transduced with empty vector control or CEACAM1-4L (4L) and analyzed for CEACAM1 and IgM expression by flow cytometry. Representative FACS plots show CEACAM1 or IgM expression from two independent experiments.

**
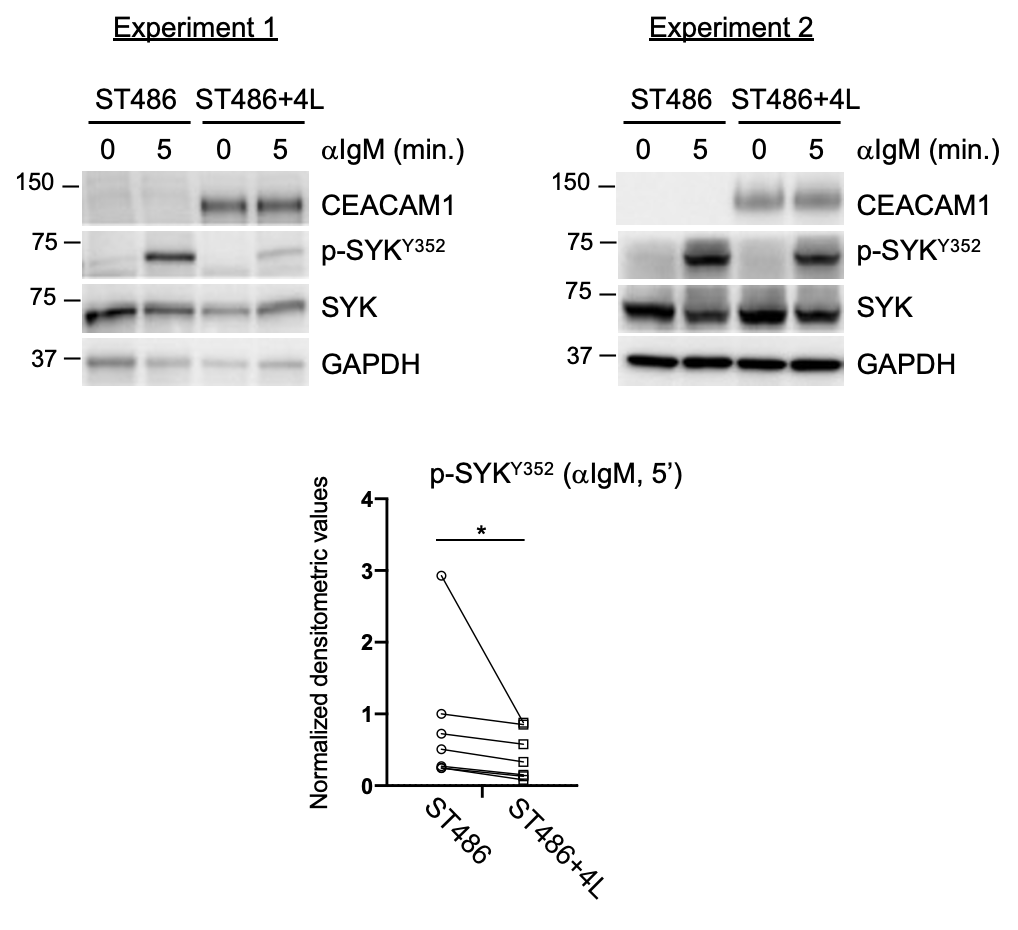
**

**Supplementary Fig. 12: Effects of CEACAM1 expression on BCR signaling in ST486 cells.** *Top panels,* ST486 cells were transduced with control or CEACAM1-4L followed by stimulation with 2 μg/ml of anti-IgM antibody for 5 min and immunoblotting with indicated antibodies. Shown are two representative experiments. *Bottom panel,* Quantitation of GAPDH-normalized p-SYK^Y352^ immunoblot densitometric values at 5 min of anti-IgM stimulation of indicated cells. Connecting lines depict direct comparison between the two indicated samples from seven independent experiments. * P<0.05 by a two-sided Wilcoxon matched-pairs signed rank test.


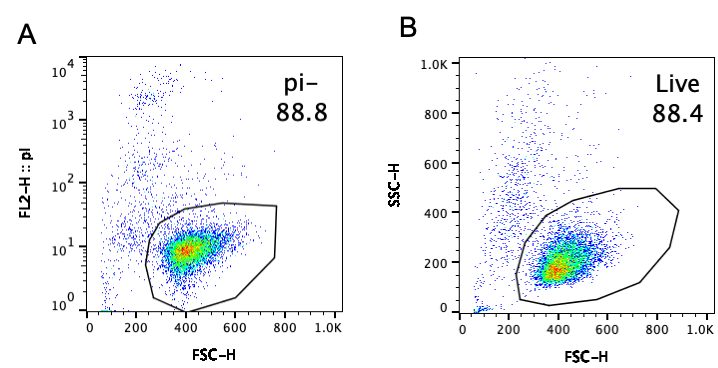


**Supplementary Fig. 13:** Gating schemes for live cells. Representative FACS plots show the live cell gates using propidium iodide-negative (pi^-^) population (A) for FACS panels in Fig. 1j, Fig. 2e, Fig. 3h, Supplementary Fig. 3a,c, Supplementary Fig. 4a, and Supplementary Fig. 6c or FSC/SSC live-cell gating (B) for Supplementary Fig. 11.
