# Supplementary material for: CEACAM1 as a mediator of B-cell receptor signaling in mantle cell lymphoma
Source: Nat Commun. 2025 May 29;16:4967. doi: 10.1038/s41467-025-60208-3 (PMC12120064; doi:10.1038/s41467-025-60208-3)
Supplement: Supplementary file 2 — Description of Additional Supplementary Information [file 41467_2025_60208_MOESM2_ESM.docx]

**Supplementary Data legends**

Supplementary Data 1: JEKO-1 CRISPR screen results

Supplementary Data 2: Prognostic predictors

Supplementary Data 3: Filtered genes shown in Fig. 1a

Supplementary Data 4: MCL PDX samples (75% male, 25% female; age range: 63-81 years old).

Supplementary Data 5: Primer sequences for amplifying sgRNA library and NGS.

Supplementary Data 6: Primer sequences for cloning sgRNA.

Supplementary Data 7: shRNA sequences.

Supplementary Data 8: Reagents, cells, animals, and software

Supplementary Data 9: Primer sequences for cloning of CEACAM1 expression vectors.

Supplementary Software: Quantitative image analysis scripts
