# Supplementary material for: CEACAM1 as a mediator of B-cell receptor signaling in mantle cell lymphoma
Source: Nat Commun. 2025 May 29;16:4967. doi: 10.1038/s41467-025-60208-3 (PMC12120064; doi:10.1038/s41467-025-60208-3)
Supplement: Supplementary file 12 — Supplementary Software [file 41467_2025_60208_MOESM12_ESM.pdf]

Supplementary Software. ***Quantitative image analysis scripts***

STEP 1 SCRIPT

```
// Script sets the image type, clears out any objects, creates a box around the entire image,
// searches it for cells using the DAPI channel, then looks within all cells for any channel 1
// regions above a manually determined threshold
setImageType('FLUORESCENCE');
clearAllObjects()
createSelectAllObject(true);
runPlugin('qupath.imagej.detect.cells.PositiveCellDetection', '{"detectionImage": "Ch1-T4", "requestedPixelSizeMicrons": 0.5, "backgroundRadiusMicrons": 0.0, "medianRadiusMicrons": 1.0, "sigmaMicrons": 2.0, "minAreaMicrons": 40.0, "maxAreaMicrons": 400.0, "threshold": 400.0, "watershedPostProcess": true, "cellExpansionMicrons": 5.0, "includeNuclei": true, "smoothBoundaries": true, "makeMeasurements": true, "thresholdCompartment": "Nucleus: Ch1-T4 mean", "thresholdPositive1": 3500.0, "thresholdPositive2": 200.0, "thresholdPositive3": 300.0, "singleThreshold": true}');
selectAnnotations();
runPlugin('qupath.imagej.detect.cells.SubcellularDetection', '{"detection[Channel 1]": 300.0, "detection[Channel 2]": -1.0, "doSmoothing": false, "splitByIntensity": false, "splitByShape": false, "spotSizeMicrons": 1.0, "minSpotSizeMicrons": 0.1, "maxSpotSizeMicrons": 2.0, "includeClusters": true}');
//Manually remove any artifacts, then run "Subcellular_Summaries.groovy"
```

STEP 2 SCRIPT

```
// Script to find average intensity of spots in cells
// Cell expansion in QuPath is blind and fixed to a certain distance,
// so a threshold was set to eliminate empty background from reducing the mean or median intensity per cell.

//Test detections for another channel.
//selectAnnotations()
//runPlugin('qupath.imagej.detect.cells.SubcellularDetection', '{"detection[Channel 1]": -1.0, "detection[Channel 2]": 1200.0, "doSmoothing": false, "splitByIntensity": false, "splitByShape": true, "spotSizeMicrons": 1.0, "minSpotSizeMicrons": 0.5, "maxSpotSizeMicrons": 2.0, "includeClusters": true}');


hierarchy = getCurrentHierarchy()
```

```
//The primary part of the script that calculates the mean intensity of the positive parts of the estimated cell area
for (annotation in getAnnotationObjects()){
    //Collect all of the cells which are children of the box around the image
    totalCells = annotation.getChildObjects()
    runningTotalArea = 0
    runningTotalIntensity = 0
    totalCells.each{
        //For each cell, collect information from all child subcellular objects
        subCellArea = 0
        subCellTotalIntensity = 0
        it.getChildObjects().each{c->

            subCellTotalIntensity += measurement(c, "Subcellular cluster: Channel 1: Area")*measurement(c, "Subcellular cluster: Channel 1: Mean channel intensity")
            subCellArea += measurement(c, "Subcellular cluster: Channel 1: Area")
        }
        //Add this information to the running total for all cells
        runningTotalArea+=subCellArea
        runningTotalIntensity += subCellTotalIntensity
        //Add a measurement to the cell with the total intensity from all subcellular objects
        it.getMeasurementList().putMeasurement("Intensity Sum", subCellTotalIntensity)
        //prevent divide by zero errors and report the average intensity for the cell
        if(subCellArea){
            it.getMeasurementList().putMeasurement("Intensity Mean", subCellTotalIntensity/subCellArea)
        }else{it.getMeasurementList().putMeasurement("Intensity Mean", 0)}
    }
    //Once all cells are processed, calculate the mean intensity across all cells.
    annotation.getMeasurementList().putMeasurement("Average positive area", runningTotalArea/totalCells.size())
    annotation.getMeasurementList().putMeasurement("Average total intensity per cell", runningTotalIntensity/totalCells.size())
}

//Uncomment these lines to remove the subcellular detections. Only do this after the accuracy of the subcellular detections has been verified!

//selectAnnotations()
//runPlugin('qupath.imagej.detect.cells.SubcellularDetection', '{"detection[Channel 1]": -1.0,  "detection[Channel 2]": -1.0,  "doSmoothing": false,  "splitByIntensity": false,  "splitByShape": true,  "spotSizeMicrons": 1.0,  "minSpotSizeMicrons": 0.5,  "maxSpotSizeMicrons": 2.0,  "includeClusters": true}');

println("Exporting results")
```

//The rest of the script looks complicated, but is simply to export the results as a text file per image, and then combine those into a single text file.
//Code source: https://petebankhead.github.io/qupath/scripting/2018/03/04/script-annotation-export.html

```
def name = getProjectEntry().getImageName() + '.txt'
def path = buildFilePath(PROJECT_BASE_DIR, 'annotation results')
mkdirs(path)
path = buildFilePath(path, name)
saveAnnotationMeasurements(path)
print 'Results exported to ' + path
path = buildFilePath(PROJECT_BASE_DIR, 'detection results')
mkdirs(path)
path = buildFilePath(path, name)
saveDetectionMeasurements(path)
print 'Results exported to ' + path
/**
 * Script to combine results tables exported by QuPath.
 *
 * This is particularly intended to deal with the fact that results tables of annotations can produce results
 * with different column names, numbers and orders - making them awkward to combine later manually.
 *
 * It prompts for a directory containing exported text files, and then writes a new file in the same directory.
 * The name of the new file can be modified - see the first lines below.
 *
 * Note: This hasn't been tested very extensively - please check the results carefully, and report any problems so they
 * can be fixed!
 *
 * @author Pete Bankhead
 */


// Some parameters you might want to change...
String ext = '.txt' // File extension to search for
String delimiter = '\t' // Use tab-delimiter (this is for the *input*, not the output)
String outputName = 'Combined_results.txt' // Name to use for output; use .csv if you really want comma separators

// Prompt for directory containing the results
//def dirResults = QuPathGUI.getSharedDialogHelper().promptForDirectory()
def dirResults = new File(buildFilePath(PROJECT_BASE_DIR, 'annotation results'))
```

```
if (dirResults == null)
    return
def fileResults = new File(dirResults, outputName)

// Get a list of all the files to merge
def files = dirResults.listFiles({
    File f -> f.isFile() &&
            f.getName().toLowerCase().endsWith(ext) &&
            f.getName() != outputName} as FileFilter)
if (files.size() <= 1) {
    print 'At least two results files needed to merge!'
    return
} else
    print 'Will try to merge ' + files.size() + ' files'

// Represent final results as a 'list of maps'
def results = new ArrayList<Map<String, String>>()

// Store all column names that we see - not all files necessarily have all columns
def allColumns = new LinkedHashSet<String>()
allColumns.add('File name')

// Loop through the files
for (file in files) {
    // Check if we have anything to read
    def lines = file.readLines()
    if (lines.size() <= 1) {
        print 'No results found in ' + file
        continue
    }
    // Get the header columns
    def iter = lines.iterator()
    def columns = iter.next().split(delimiter)
    allColumns.addAll(columns)
    // Create the entries
    while (iter.hasNext()) {
        def line = iter.next()
        if (line.isEmpty())
            continue
        def map = ['File name': file.getName()]
        def values = line.split(delimiter)
        // Check if we have the expected number of columns
        if (values.size() != columns.size()) {
            print String.format('Number of entries (%d) does not match the number of columns
(%d)!', columns.size(), values.size())
            print('I will stop processing ' + file.getName())
```

```
            break
        }
        // Store the results
        for (int i = 0; i < columns.size(); i++)
            map[columns[i]] = values[i]
        results.add(map)
    }
}

// Create a new results file - using a comma delimiter if the extension is csv
if (outputName.toLowerCase().endsWith('.csv'))
    delimiter = ','
int count = 0
fileResults.withPrintWriter {
    def header = String.join(delimiter, allColumns)
    it.println(header)
    // Add each of the results, with blank columns for missing values
    for (result in results) {
        for (column in allColumns) {
            it.print(result.getOrDefault(column, ''))
            it.print(delimiter)
        }
        it.println()
        count++
    }
}

// Success!  Hopefully...
print 'Done! ' + count + ' result(s) written to ' + fileResults.getAbsolutePath()


dirResults = new File(buildFilePath(PROJECT_BASE_DIR, 'detection results'))
if (dirResults == null)
    return
fileResults = new File(dirResults, outputName)

// Get a list of all the files to merge
files = dirResults.listFiles({
    File f -> f.isFile() &&
            f.getName().toLowerCase().endsWith(ext) &&
            f.getName() != outputName} as FileFilter)
if (files.size() <= 1) {
    print 'At least two results files needed to merge!'
    return
} else
    print 'Will try to merge ' + files.size() + ' files'
```

```
// Represent final results as a 'list of maps'
results = new ArrayList<Map<String, String>>()

// Store all column names that we see - not all files necessarily have all columns
allColumns = new LinkedHashSet<String>()
allColumns.add('File name')

// Loop through the files
for (file in files) {
    // Check if we have anything to read
    def lines = file.readLines()
    if (lines.size() <= 1) {
        print 'No results found in ' + file
        continue
    }
    // Get the header columns
    def iter = lines.iterator()
    def columns = iter.next().split(delimiter)
    allColumns.addAll(columns)
    // Create the entries
    while (iter.hasNext()) {
        def line = iter.next()
        if (line.isEmpty())
            continue
        def map = ['File name': file.getName()]
        def values = line.split(delimiter)
        // Check if we have the expected number of columns
        if (values.size() != columns.size()) {
            print String.format('Number of entries (%d) does not match the number of columns
(%d)!', columns.size(), values.size())
            print('I will stop processing ' + file.getName())
            break
        }
        // Store the results
        for (int i = 0; i < columns.size(); i++)
            map[columns[i]] = values[i]
        results.add(map)
    }
}

// Create a new results file - using a comma delimiter if the extension is csv
if (outputName.toLowerCase().endsWith('.csv'))
    delimiter = ','
count = 0
fileResults.withPrintWriter {
```

```
    header = String.join(delimiter, allColumns)
    it.println(header)
    // Add each of the results, with blank columns for missing values
    for (result in results) {
        for (column in allColumns) {
            it.print(result.getOrDefault(column, ''))
            it.print(delimiter)
        }
        it.println()
        count++
    }
}

// Success!  Hopefully...
print 'Done! ' + count + ' result(s) written to ' + fileResults.getAbsolutePath()

import qupath.lib.gui.QuPathGUI
import qupath.lib.objects.PathCellObject
```
