# Supplementary material for: CEACAM1 as a mediator of B-cell receptor signaling in mantle cell lymphoma
Source: Nat Commun. 2025 May 29;16:4967. doi: 10.1038/s41467-025-60208-3 (PMC12120064; doi:10.1038/s41467-025-60208-3)
Supplement: Supplementary file 13 — Reporting Summary [file 41467_2025_60208_MOESM13_ESM.pdf]

nature portfolio

Corresponding author(s): Vu Ngo

Last updated by author(s): May 7, 2024

Reporting Summary

Nature Portfolio wishes to improve the reproducibility of the work that we publish. This form provides structure for consistency and transparency in reporting. For further information on Nature Portfolio policies, see our Editorial Policies and the Editorial Policy Checklist.

Statistics

For all statistical analyses, confirm that the following items are present in the figure legend, table legend, main text, or Methods section.

n/a	Confirmed	
☐	☒	The exact sample size (*n*) for each experimental group/condition, given as a discrete number and unit of measurement
☐	☒	A statement on whether measurements were taken from distinct samples or whether the same sample was measured repeatedly
☐	☒	The statistical test(s) used AND whether they are one- or two-sided<br>*Only common tests should be described solely by name; describe more complex techniques in the Methods section.*
☒	☐	A description of all covariates tested
☒	☐	A description of any assumptions or corrections, such as tests of normality and adjustment for multiple comparisons
☐	☒	A full description of the statistical parameters including central tendency (e.g. means) or other basic estimates (e.g. regression coefficient) AND variation (e.g. standard deviation) or associated estimates of uncertainty (e.g. confidence intervals)
☐	☒	For null hypothesis testing, the test statistic (e.g. *F*, *t*, *r*) with confidence intervals, effect sizes, degrees of freedom and *P* value noted<br>*Give P values as exact values whenever suitable.*
☒	☐	For Bayesian analysis, information on the choice of priors and Markov chain Monte Carlo settings
☐	☒	For hierarchical and complex designs, identification of the appropriate level for tests and full reporting of outcomes
☒	☐	Estimates of effect sizes (e.g. Cohen's *d*, Pearson's *r*), indicating how they were calculated

Our web collection on statistics for biologists contains articles on many of the points above.

Software and code

Policy information about availability of computer code

Data collection	Chemiluminescence signals obtained from immunoblots were captured using the ChemiDoc Imaging System (Bio-Rad). qPCR assays to measure mRNA expression were performed using QuantStudio 12K Flex Real-Time PCR System (ThermoFisher). Data from flow cytometry-based cytotoxicity assays were acquired using FACSCalibur Analyzer (Becton Dickenson Biosciences). Fluorescent images were acquired on an LSM880 microscope (Carl Zeiss Microscopy) and were controlled with the ZEN 2.3 SP1 Black Edition software (Carl Zeiss Microscopy). Tumor growth in vivo was monitored by whole-body bioluminescence imaging using a Spectral LagoX camera (Spectral Instrument Imaging).
Data analysis	The CRISPR library screen results were analyzed using the open source MAGeCK software (http://liulab.dfci.harvard.edu/Mageck). Transcriptome data were analyzed using R Bioconductor packages for Affymetrix Oligonucleotide Arrays. Quantification of immunoblot signal intensity was analyzed using the Open-Source ImageJ software (imagej.nih.gov). qPCR data were processed using QuantStudio™ Real-Time PCR Software (Applied Biosystems). Data outputs were exported as Excel Spreadsheets. Flow cytometry data were analyzed using FlowJo version 9 software (FlowJo, LLC). Quantitative fluorescent image analysis was performed using the open source QuPath 0.2.0m6 software. The scripts for image analysis are available in Supplementary Materials S2. In vivo bioluminescence images were analyzed using Aura software (Spectral Instrument Imaging). Statistical analyses were performed using GraphPad Prism 8 (GraphPad Software Inc.)

For manuscripts utilizing custom algorithms or software that are central to the research but not yet described in published literature, software must be made available to editors and reviewers. We strongly encourage code deposition in a community repository (e.g. GitHub). See the Nature Portfolio guidelines for submitting code & software for further information.

Data

Policy information about availability of data

All manuscripts must include a data availability statement. This statement should provide the following information, where applicable:

- Accession codes, unique identifiers, or web links for publicly available datasets
- A description of any restrictions on data availability
- For clinical datasets or third party data, please ensure that the statement adheres to our policy

All data are available in the main text or in the supplementary materials.

Research involving human participants, their data, or biological material

Policy information about studies with human participants or human data. See also policy information about sex, gender (identity/presentation), and sexual orientation and race, ethnicity and racism.

Reporting on sex and gender	Not applicable
Reporting on race, ethnicity, or other socially relevant groupings	Not applicable
Population characteristics	Not applicable
Recruitment	Not applicable
Ethics oversight	The use of human biological materials in this study was approved by the City of Hope's Institutional Review Board.

Note that full information on the approval of the study protocol must also be provided in the manuscript.

Field-specific reporting

Please select the one below that is the best fit for your research. If you are not sure, read the appropriate sections before making your selection.

[x] Life sciences [ ] Behavioural & social sciences [ ] Ecological, evolutionary & environmental sciences

For a reference copy of the document with all sections, see nature.com/documents/nr-reporting-summary-flat.pdf

Life sciences study design

All studies must disclose on these points even when the disclosure is negative.

Sample size	Sample size was determined based on prior experiments and replications.
Data exclusions	No data were excluded.
Replication	All experiments were successfully replicated at least two times.
Randomization	Randomization was not relevant to this study due to its focus on signal transduction mechanisms.
Blinding	Blinding was not relevant to this study due to its focus on signal transduction mechanisms.

Reporting for specific materials, systems and methods

We require information from authors about some types of materials, experimental systems and methods used in many studies. Here, indicate whether each material, system or method listed is relevant to your study. If you are not sure if a list item applies to your research, read the appropriate section before selecting a response.

Materials & experimental systems

n/a	Involved in the study	
	x	Antibodies
	x	Eukaryotic cell lines
x		Palaeontology and archaeology
	x	Animals and other organisms
x		Clinical data
x		Dual use research of concern
x		Plants

Methods

n/a	Involved in the study	
x		ChIP-seq
	x	Flow cytometry
x		MRI-based neuroimaging

Antibodies

Antibodies used

Antibodies Source Identifier
Beta-catenin (12F7) mouse IgG1 Santa Cruz Biotechnology sc-59737
Phospho-GSK3b (S9) (D3A4) Rabbit IgG Cell Signaling Technology 9322S
GSK3-beta (D5C5Z) Rabbit IgG 12456S
CD79A Rabbit IgG Cell Signaling Technology 3351
Phospho-CD79A (Tyr188) Rabbit IgG Cell Signaling Technology 5173
CD79B (B29/123) Mouse IgG2b Santa Cruz Biotechnology sc-53210
LYN Rabbit IgG Cell Signaling Technology 2732
Phospho-Src (Tyr416) Rabbit IgG Cell Signaling Technology 2101
Phospho-SYK (Tyr352) Rabbit IgG Cell Signaling Technology 2701
Phospho-SYK (Tyr525/526) Rabbit IgG Cell Signaling Technology 2711
Phospho-PLCγ1 (Tyr783) Rabbit IgG Cell Signaling Technology 14008
Phospho-p44/42 MAPK (Erk1/2) (Thr202/Tyr204) Rabbit IgG Cell Signaling Technology 4376
Phospho-Akt (Ser473) Rabbit IgG Cell Signaling Technology 9271
SYK Rabbit IgG Cell Signaling Technology 2712
PLCγ1Rabbit IgG Cell Signaling Technology 2822
p44/42 MAPK (Erk1/2) (137F5) Rabbit IgG Cell Signaling Technology 4695
AKT Rabbit IgG Cell Signaling Technology 9272
CEACAM1 (E-1) Mouse IgG2b Santa Cruz Biotechnology sc-166453
LYN (H-6) Mouse IgG2a Santa Cruz Biotechnology sc-7274
Filamin 1 (FLMN01) Mouse IgG1 Santa Cruz Biotechnology sc-58763
GAPDH (6C5) Mouse IgG1 Santa Cruz Biotechnology sc-32233
CEACAM1 [EPR4049] Rabbit IgG Abcam ab108397
SHP-1 (D-11) Mouse IgG3 Santa Cruz Biotechnology sc-7289
Goat anti-Mouse IgG2a, Alexa Fluor™ 555 Invitrogen A-21137
Goat anti-Mouse IgG1, Alexa Fluor™ 647 Invitrogen A-21240
AffiniPure F(ab')$_2$ fragment donkey anti-Human IgM Jackson ImmunoResearch 709-006-073
Rabbit IgG (H+L) Invitrogen A-21206
Mouse IgG2b-UNLB (A-1) isotype control SouthernBiotech 0104-01
HA-Tag (C29F4) rabbit mAb Cell Signaling Technology 3724
HA-Tag (6E2) Mouse mAb Cell Signaling Technology 2367
AffiniPure Donkey Anti-Mouse IgG (H+L) Jackson ImmunoResearch 715-005-150
AffiniPure Donkey Anti-Rabbit IgG (H+L) Jackson ImmunoResearch 711-005-152
SHP-1 Monoclonal Antibody (1SH01 (11D7C8H5)) ThermoFisher Scientific MA5-11669
mCEACAM1 Invitrogen 14-0661-82
p-Y (4G10) EMD Millipore 05-321
Goat Anti-Mouse IgG2a, Human ads-HRP SouthernBiotech 1080-05
Goat Anti-Mouse IgG2b, Human ads-HRP SouthernBiotech 1090-05
Goat Anti-Mouse IgG1, Human ads-HRP SouthernBiotech 1070-05
Goat Anti-Mouse IgG3, Human ads-HRP SouthernBiotech 1100-05
Goat Anti-Rabbit Ig, Human ads-HRP SouthernBiotech 4010-05

Validation

Validation of CEACAM1 antibodies include gene knockout or knockdown, overexpression, or using CEACAM1 knockout mice. For other primary antibodies, validation was based on the citations and long-term establishment of the antibodies in the field.

Eukaryotic cell lines

Policy information about cell lines and Sex and Gender in Research

Cell line source(s)

Cell lines	Source	Sex
JEKO-1	ATCC	Female
Z-138	ATCC	Male
MINO	ATCC	Male

REC-1 ATCC Male
MAVER-1 ATCC Male
SP-53 Dr. Louis Staudt Female
SUDHL-4 Dr. Louis Staudt Male
SUDHL-6 Dr. Louis Staudt Male
OCI-LY10 Dr. Louis Staudt Female
HBL-1 Dr. Louis Staudt Male
RAMOS Dr. Louis Staudt Male
K1718 Dr. Louis Staudt Male
SSK41 Dr. Louis Staudt Unspecified
H-929 Dr. Louis Staudt Female
MCL 2 COH tissue bank Unspecified
MCL 3 COH tissue bank Unspecified
MCL 4 COH tissue bank Unspecified
MCL 6 COH tissue bank Unspecified
MCL 9 COH tissue bank Unspecified
MCL 12 COH tissue bank Unspecified
MCL PDX 4 ProXe.org Male
MCL PDX 5 ProXe.org Male
MCL PDX 7 ProXe.org Male
MCL PDX 9 ProXe.org Female

Authentication	All cell lines were authenticated by commercial DNA fingerprinting (ATCC and Laragen).
Mycoplasma contamination	Most cell lines were tested negative for mycoplasma.
Commonly misidentified lines (See ICLAC register)	No commonly misidentified cell lines were used in this study.

Animals and other research organisms

Policy information about studies involving animals; ARRIVE guidelines recommended for reporting animal research, and Sex and Gender in Research

Laboratory animals	Ceacam1–/– mice and Eu-SOX11/CCND1 mice were maintained on the C57BL/6 background. Xenografts were performed using NOD.Cg-PrkdcscidIl2rgtm1Wjl/SzJ (NSG) mice.
Wild animals	Not applicable
Reporting on sex	Not applicable
Field-collected samples	Not applicable
Ethics oversight	The use of animals in this study was performed under City of Hope's IACUC-approved protocol #11046 and #22054.

Note that full information on the approval of the study protocol must also be provided in the manuscript.

Plants

Seed stocks	Not applicable
Novel plant genotypes	Not applicable
Authentication	Not applicable

Flow Cytometry

Plots

Confirm that:

☒ The axis labels state the marker and fluorochrome used (e.g. CD4-FITC).

☒ The axis scales are clearly visible. Include numbers along axes only for bottom left plot of group (a 'group' is an analysis of identical markers).

☒ All plots are contour plots with outliers or pseudocolor plots.

☒ A numerical value for number of cells or percentage (with statistics) is provided.

Methodology

Sample preparation	Cells were resuspended in FACS staining buffer (2% FBS and 0.05% sodium azide in 1X Phosphate buffered saline) together with diluted fluorphore-conjugated antibodies and stained for 20 minutes on ice followed by washing once in 1x PBS and resuspended in FACS buffer for flow cytometry analysis.
Instrument	FACSCalibur Analyzer (Becton Dickenson Biosciences)
Software	FlowJo version 9 software (FlowJo, LLC)
Cell population abundance	No post-sort cells are used in this study.
Gating strategy	Provided in Supplementary Fig. 1.

☒ Tick this box to confirm that a figure exemplifying the gating strategy is provided in the Supplementary Information.
